# Characterization of a New *Staphylococcus aureus* *Kayvirus* Harboring a Lysin Active against Biofilms

**DOI:** 10.3390/v10040182

**Published:** 2018-04-07

**Authors:** Luís D. R. Melo, Ana Brandão, Ergun Akturk, Silvio B. Santos, Joana Azeredo

**Affiliations:** LIBRO—Laboratório de Investigação em Biofilmes Rosário Oliveira, Centre of Biological Engineering, University of Minho, Campus de Gualtar, 4700-057, Braga, Portugal; lmelo@deb.uminho.pt (L.D.R.M.); anacatarinabr@hotmail.com (A.B.); ergun.akturk@ceb.uminho.pt (E.A.); silviosantos@ceb.uminho.pt (S.B.S.)

**Keywords:** *Staphylococcus aureus*, *Kayvirus*, bacteriophage, endolysin, biofilms

## Abstract

*Staphylococcus aureus* is one of the most relevant opportunistic pathogens involved in many biofilm-associated diseases, and is a major cause of nosocomial infections, mainly due to the increasing prevalence of multidrug-resistant strains. Consequently, alternative methods to eradicate the pathogen are urgent. It has been previously shown that polyvalent staphylococcal kayviruses and their derived endolysins are excellent candidates for therapy. Here we present the characterization of a new bacteriophage: vB_SauM-LM12 (LM12). LM12 has a broad host range (>90%; 56 strains tested), and is active against several MRSA strains. The genome of LM12 is composed of a dsDNA molecule with 143,625 bp, with average GC content of 30.25% and codes for 227 Coding Sequences (CDSs). Bioinformatics analysis did not identify any gene encoding virulence factors, toxins, or antibiotic resistance determinants. Antibiofilm assays have shown that this phage significantly reduced the number of viable cells (less than one order of magnitude). Moreover, the encoded endolysin also showed activity against biofilms, with a consistent biomass reduction during prolonged periods of treatment (of about one order of magnitude). Interestingly, the endolysin was shown to be much more active against stationary-phase cells and suspended biofilm cells than against intact and scraped biofilms, suggesting that cellular aggregates protected by the biofilm matrix reduced protein activity. Both phage LM12 and its endolysin seem to have a strong antimicrobial effect and broad host range against *S. aureus*, suggesting their potential to treat *S. aureus* biofilm infections.

## 1. Introduction

Currently, the development of bacterial resistance to antibiotics has become a global concern. Very recently, the World Health Organization (WHO) published a list of priority pathogens resistant to antibiotics, encouraging the scientific community and pharmaceutical industries to focus on the development of new antimicrobials to combat antimicrobial-resistant (AMR) pathogens [1]. In the referred report, methicillin-resistant *Staphylococcus aureus* (MRSA), vancomycin-intermediate *S. aureus* (VISA), and vancomycin-resistant *S. aureus* (VRSA) were considered of high importance [1].

Antibiotic-resistant *S. aureus* is well-established in both community and healthcare settings, being one the most frequently isolated pathogens in hospital-associated infections (HAI) [2]. This microorganism is a resourceful pathogen that can cause a wide range of diseases, from food poisoning to life-threatening diseases such as pneumonia, infective endocarditis, or sepsis [3].

Recent evidence has revealed that *S. aureus* infections are one of the main causes of hospital infections, leading to increasing rates of morbidity and mortality, which ultimately increase health care costs [4,5]. The severity of *S. aureus* infections is mostly related to its intrinsic ability to acquire and express antibiotic-resistance genes. Presently, *S. aureus* has acquired a resistance to practically all antibiotics [6]. Moreover, its ability to adhere to host tissues and evade human host defenses, namely by the formation of thick biofilms, increases the difficulty in treating this pathogen [7]. Indeed, biofilm cells are described as being more tolerant to antibiotics than planktonic cells, mainly due to the antibiotics’ difficulty penetrating the complex biofilm matrix [8]. Moreover, the slow growth of biofilm cells limits therapeutic success, as several antibiotics can only target active cells.

Bacteriophages, also known as phages, are natural bactericidal agents that specifically lyse bacteria. Due to their bacterial host specificity and bacteriolytic activity against antibiotic-resistant strains, the use of phage therapy has been suggested as a valuable approach to control numerous pathogenic bacteria, namely *S. aureus*. During the last decade, a huge importance has been given to the study of staphylococcal phages, namely their genomes. Although the majority are temperate siphoviruses, staphylococcal myoviruses have been described as very promising for therapy purposes [9,10]. Moreover, staphylococcal lytic phages have demonstrated their in-vitro potential as anti-biofilm agents on food matrices and on clinical models, controlling in-vitro biofilms [11,12,13].

In addition, phage-encoded cell-wall-degrading enzymes, named endolysins, have been proposed to be potent antibacterial agents [14]. These peptidoglycan hydrolases evolved to rapidly break down the bacterial cell wall, thereby allowing the release of phage progeny. When endolysins were expressed heterologously and added to the cells from the outside, they displayed a high bacteriolytic effect in Gram-positive cells.

In this study, we report the isolation of a new *Kayvirus* phage, named vB_SauM-LM12 (LM12). This phage was characterized morphologically and genomically, revealing suitable properties for therapy. We also purified and characterized the staphylococcal endolysin E-LM12, derived from the phage LM12, and demonstrated the former’s efficacy as a valuable anti-biofilm agent.

## 2. Materials and Methods

### 2.1. Bacterial Strains and Culture Conditions

*S. aureus* collection strains were obtained from American Type Culture Collection (ATCC) and Spanish Type Culture Collection (CECT). Additional *S. aureus* clinical isolates were obtained from the Hospital Strain Collection (Braga, Portugal). To complete the phage and endolysin lytic spectrum, additional staphylococcal strains, and other Gram-positive species from our collection were used. This accounts to a total of 39 strains used (Table 1), including 29 *S. aureus*, 7 non-*S. aureus* staphylococcal strains, and one representative of *Enterococcus faecalis*, *Enterococcus faecium*, and *Listeria monocytogenes*. All strains were grown in Tryptic Soy Broth (TSB, VWR Chemicals, Randor, PA, USA), Tryptic Soy Agar (TSA; VWR Chemicals) or in TSA soft overlays (TSB with 0.6% agar) at 37 °C.

### 2.2. Phage Isolation

Phages were isolated from effluent samples of different wastewater treatment plants (WWTP): Paradela (Vila Verde), Frossos (Braga). The sample enrichment method was performed to isolate phages [16]. Briefly, 20 mL of the effluent were mixed with 20 mL double-strength TSB and with 50 μL of each of the exponentially grown *S. aureus* strains. The resulting suspensions were incubated at 37 °C and 120 rpm (BIOSAN ES-20/60, Riga, Latvia) over 24 h. Suspensions were further centrifuged (10 min, 10,000× *g*, 4 °C), and the resulting supernatants were filtered through a 0.22-μm PES membrane (GE Healthcare, Little Chalfont, UK).

The presence of phages was checked by performing spot assays on bacterial lawns. Plates were further incubated at 37 °C, for 16–18 h, and the presence of inhibition halos assessed. When present, the inhibition halos where submitted to repeated picking, until single-plaque morphology was observed and ten plaques of each isolated phage were measured and characterized.

### 2.3. Phage Production

Phage particles were produced using the double agar layer method as described before [17]. Briefly, 100 μL of a phage suspension at 10^6^ PFU·mL^−1^ was spread on a *S. aureus* Sa12 lawn, using a paper strip. After 16–18 h of incubation at 37 °C, full lysis was checked. Plates were agitated at 120 rpm in an orbital shaker (BIOSAN PSU-10i) for 24 h at 4 °C, with 3 mL of an SM Buffer (100 mM NaCl, 8 mM MgSO_4_, 50 mM Tris/HCl (pH 7.5)) to resuspend the phage particles. The liquid phase was collected and centrifuged (10 min, 10,000× *g*, 4 °C).

Phages were concentrated, as described by Sambrook and Russel [18]. First, the phage lysate was concentrated with NaCl (5.84% *w*/*v*) and PEG 8000 (10% *w*/*v*), then purified with chloroform (1:4 *v*/*v*). After filtration through a 0.22-μm PES membrane, the samples were stored at 4 °C until further use.

### 2.4. Electron Microscopy

Phage particles were sedimented by centrifugation (25,000× *g*, 60 min, 4 °C) using a ScanSpeed 1730R centrifuge (Labogene, Lillerød, Denmark). The pellet was further washed in tap water by repeating the centrifugation step. Subsequently, phage suspension was deposited on copper grids with a carbon-coated Formvar carbon film on a 200 square mesh nickel grid, stained with 2% uranyl acetate (pH 4.0) and examined using a Jeol JEM 1400 transmission electron microscope (TEM) (Tokyo, Japan).

### 2.5. Lytic Spectra and Efficiency of Plating

The host range specificity and lysis efficiency of the isolated phage was screened against all strains listed in Table 1. Bacterial lawns were made on TSA plates by adding 100 μL of exponential-phase cell cultures of each strain. The bacterial lawns were spotted with 10 μL drops of serial 10-fold dilutions of the phage solution. After 16–18 h incubation at 37 °C, results were observed and scored. The relative efficiency of plating (EOP) was calculated as the ratio between the phage titer (PFU/mL) obtained in each isolate and the one obtained in the propagating host.

### 2.6. DNA Isolation, Genome Sequencing, and in Silico Analysis

Phage DNA was extracted essentially as described before [17]. Briefly, purified phages were treated with 0.016% (*v*/*v*) L1 buffer (300 mM NaCl, 100 mM Tris/HCl (pH 7.5), 10 mM EDTA, 0.2 mg BSA mL^−1^, 20 mg RNase A mL^−1^ (Sigma, Saint Louis, MO, USA), 6 mg DNase I mL^−1^ (Sigma)) for 2 h at 37 °C, and the enzymes were further thermally inactivated for 30 min at 65 °C. Proteins were further digested with 50 μg proteinase K mL^−1^, 20 mM EDTA and 1% SDS, for 18 h at 56 °C. This was followed by phenol, phenol:chloroform (1:1, *v*/*v*) and chloroform extractions. DNA was then precipitated with isopropanol and 3 M sodium acetate (pH 4.6), centrifuged (15 min, 7600× *g*, 4 °C), and the pellet air-dried and resuspended in nuclease-free water (Cleaver Scientific, Rugby, UK).

Afterwards, the phage genome was sequenced with an Illumina MiSeq platform conducted in Nucleomics Core (VIB, Leuven, Belgium). LM12 DNA was mixed with another non-homologous phage (at equimolar ratio) and subjected to quality controls using an Agilent Bioanalyzer (Santa Clara, CA, USA). DNA library preparations were made by a custom kit (Nextera XT sample prep, Illumina, San Diego, CA, USA) to generate an average insert size of 500 bp. All DNA library preparations were pooled together and sequenced using 150-bp unpaired reads. After processing, reads were trimmed to remove adapters, contaminations, or low-quality sequences. Contigs were assembled, with a relatively homogenous coverage, with the CLC genomics Workbench version 7 (CLC Bio, Aarhus, Denmark), using the de novo assembly algorithm and manual inspection.

The genome was firstly annotated using MyRAST algorithm [19], and was further manually inspected for potential alternative start codons or for the presence of non-annotated CDSs using Geneious 9.1.4 (Biomatters Ltd., Auckland, New Zealand). Functions of the gene products were searched with BLASTp [20] and Pfam [21] programs (*E*-value ≤ 10^−5^). The presence of transmembrane domains was checked using TMHMM [22] and Phobius [18], and membrane proteins were annotated when both tools were in concordance. Protein parameters (molecular weight and isoelectric point) were determined using ExPASy Compute pI/Mw [23]. Transfer RNAs (tRNAs) were scanned using tRNAscan-SE [24] and ARAGORN [18].

Promoter regions were determined using PromoterHunter from the phiSITE database [25] and checked manually. ARNold [26] was used to predict rho-independent terminators and the energy was calculated using Mfold [27]. The DNA homology comparisons between phages were conducted with progressiveMauve [28].

The phylogenetic analysis of homologous phages was performed on Geneious 9.1.4. For phylogenetic purposes, the LM12 genome was aligned with 27 other staphylococcal phage genomes using the MAFFT Alignment on Geneious. The resulting alignment was used to build a phylogenetic tree using a Maximum-Likelihood phylogenetic (PHYML) algorithm with a bootstrapping of 100.

### 2.7. Expression Plasmid Construction

Primers containing the restriction cloning sites of NdeI/XhoI (underlined) were designed to amplify the LM12 putative lysin (forward CCGCCG CATATG GAATTCATGGCTAAGACTCAAGCAGAAATAAATAAA, and reverse CCGCCG CTCGAG TTAACCTTTGAATACACCCCAGG) by PCR with Phusion Polymerase (ThermoFisher, Waltham, MA, USA). The PCR product was digested with proper endonucleases (FastDigest—ThermoFisher) and ligated with T4 DNA ligase (New England Biolabs, Ipswich, MA, USA), according to the manufacturer’s protocols, into pET28a (Novagen, Merck Millipore, Burlington, MA, USA), resulting in the plasmid pET28a–LM12 encoding the lysin with a C-terminal His-tag. The resulting plasmid was propagated and maintained in *E. coli* TOP10. The correct insertion of the lysin gene into the plasmid was confirmed by Sanger sequencing (GATC Biotech, Konstanz, Germany).

### 2.8. Recombinant Protein Production and Purification

*E. coli* BL21(DE3) cells transformed with the recombinant plasmid were grown at 37 °C in an LB medium supplemented with the appropriate antibiotic (50 μg/mL of kanamycin), until an OD_620nm_ of 0.550 and recombinant protein expression was induced by IPTG (1 mM final concentration) overnight at 16 °C. The cells were recovered by centrifugation, resuspended in lysis buffer (20 mM Na_2_H_2_PO_4_, 500 mM sodium chloride, 10 mM imidazole, pH 7.4), and disrupted to release the expressed protein. Cell disruption was accomplished by three thaw-freezing cycles (from −80 °C to room temperature) followed by a 5-min sonication (Cole-Parmer Ultrasonic Processor, Vernon Hills, IL, USA) for 10 cycles (30 s pulse, 30 s pause) at 40% amplitude. Soluble cell-free extracts were collected by centrifugation, filtered, and loaded on a 1 mL Ni-NTA agarose stacked in a Polypropylene column (Qiagen, Hilden, Germany), previously equilibrated with 10 mM imidazole, making use of the protein N-terminal His-tag. The washing step was performed using protein-dependent imidazole concentrations (lysis buffer supplemented with 20 mM imidazole in the first wash and 40 mM imidazole in the second) and elution, carried out with 300 mM imidazole in lysis buffer.

The purified protein was analyzed by SDS-PAGE using a 12% (*w*/*v*) acrylamide gel, followed by BlueSafe staining (NZYTech, Lisbon, Portugal). The eluted protein was concentrated and dialyzed against 20 mM HEPES pH 8.0 using the centrifugal filters Amicon Ultra—0.5 mL (Merck Millipore) and stored at 4 °C.

### 2.9. Activity on Planktonic Cells

The activity of E-LM12 lysin against planktonic cells in both exponential and stationary phases was performed as described before [29]. In brief, to obtain a suspension of stationary phase cells, *S. aureus* was grown for 48 h at 37 °C and 120 rpm. Cell suspensions were diluted with spent medium (centrifuged medium used to grow the cells, and to avoid regrowth of the cells) to obtain an optical density at 600 nm (OD_600nm_) of approximately 0.4 (~5 × 10^8^ colony forming units (CFU)·mL^−1^). Exponentially growing cells were obtained by inoculating 10 mL of fresh TSB with 100 μL of the overnight grown culture, and allowing bacteria to grow until OD_600nm_ reached approximately 0.4.

Cells were pelleted by centrifugation and further resuspended in 8 μM of endolysin. Samples were taken at 2 h, 6 h, and 24 h post-contact with the lysin. The number of cultivable cells (CFU·mL^−1^) was quantified using the microdrop method (10 μL drops of 10-fold dilutions of the suspension are spotted on agar medium petri plates and the number of CFUs recorded), and the antibacterial activity assessed as the relative inactivation in logarithmic units (log10 (*N*_0_/*N_i_*); *N*_0_ is the number of untreated cells in the negative control, and *N_i_* is the number of treated cells counted after incubation). Three independent experiments were performed in triplicate. Control experiments were performed by adding 20 mM HEPES, pH 8.0 buffer instead of endolysin suspension.

### 2.10. Biofilm Formation

Biofilm formation was performed by inoculating one *S. aureus* colony in TSB (10 mL), and incubating for 16 h in an orbital shaker (120 rpm, BIOSAN) at 37 °C. To establish mature biofilms, 2 μL of the starter culture were transferred into 96-well polystyrene plates (Orange Scientific, Braine-l’Alleud, Belgium) containing 198 μL of TSB supplemented with 1% filtered glucose (*w*/*v*) (TSBG). The plates were incubated for 24 h in an orbital shaker incubator (120 rpm, BIOSAN ES-20/60) at 37 °C. Biofilms were washed twice more with saline solution. After scraping, samples were sonicated for 10 s at 30% amplitude to eliminate clusters [30], and the number of cultivable cells determined using the microdrop method. Three independent experiments were performed in triplicate.

### 2.11. Biofilm Challenge

Twenty-four hour biofilms were infected as previously described [29], with some modifications. Briefly, biofilm supernatants were removed, and the biofilms were washed twice with saline solution. Thereafter, 200 μL of phage (Multiplicity of infection of 1) or endolysin (8 μM) were added to each well. Microplates were incubated at 37 °C and 120 rpm, and samples were taken at 2 h, 6 h, and 24 h post-infection. Samples were sonicated for 10 s at 30%, and the number of cultivable cells determined using the microdrop method. Three independent experiments were performed in triplicate. Control experiments were performed by adding SM buffer instead of phage suspension, or 20 mM HEPES and pH 8.0 instead of endolysin.

### 2.12. Minimal Inhibitory Concentration Assay

A classical microdilution broth method was used to determine E-LM12 minimal inhibitory concentration (MIC), as previously described [31,32]. All MIC values represent three assays, with three replicates in each assay.

### 2.13. Infection of Scraped Biofilm Cells

To assess lysin activity against disrupted biofilms, 24 h biofilms were washed twice with saline solution, and the biofilm three-dimensional (3D) structure was disturbed by a slight manual scraping. Biofilm suspension was further challenged with the endolysin, using the conditions described for biofilm challenge. Control experiments were performed by adding HEPES buffer instead of endolysin suspension. Three independent experiments were performed in duplicate.

### 2.14. Infection of Suspended Biofilm Cells

The endolysin activity on biofilm cells was tested by manually scraping double-washed 24 h biofilms until their full detachment from the surfaces. Samples were homogenized by vortexing, to allow each matrix to detach from the biofilm cells and be further challenged with the endolysin, using reaching the conditions described for the biofilm challenge. Control experiments were performed by adding HEPES buffer instead of endolysin suspension. Three independent experiments were performed in duplicate.

### 2.15. Statistical Analysis

The assays were compared using two-way ANOVA and Sidak post-test, using Prism 6 (GraphPad, La Jolla, CA, USA). Means and standard deviations (SD) were calculated. Differences among conditions were considered statistically significant when *p* < 0.01.

## 3. Results

### 3.1. LM12 Is a New S. aureus-Infecting Phage

**Figure 1:**
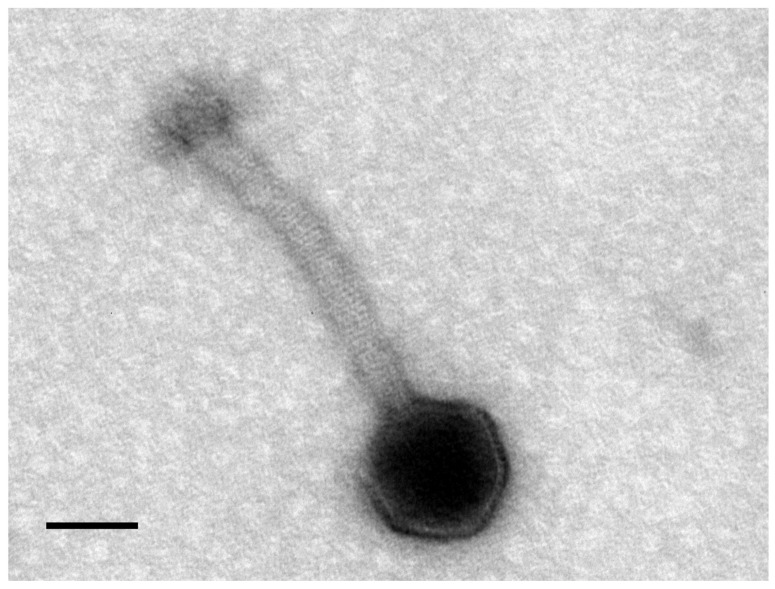
Morphology of LM12. Transmission electron microscopic image of *Staphylococcus* myovirus LM12 negatively stained with 2% (*w*/*v*) uranyl acetate. Scale bar represents 50 nm.

Twenty-six MRSA clinical strains were used for phage enrichment, using wastewater treatment plant sewage samples as phage sources. Although no phages were detected in the Frossos effluents, when using Paradela raw sewage a phage vB_SauM_LM12 (LM12) was isolated, and its plaque morphology was characterized by clear and uniform small plaques (0.5 mm in diameter) on the host strain (*n* = 10). According to TEM micrographs, LM12 belongs to the *Myoviridae* family (Figure 1). Phage particles are composed by an icosahedral head, 83 nm in diameter, and a contractile tail of 168 × 20 nm (*n* = 5). Furthermore, phage particles are composed of a neck, conspicuous transverse tail striations, a baseplate, and terminal bulbous spikes.

### 3.2. LM12 Is a Polyvalent Phage with a Wide Host Lytic Range among Clinical Isolates

A collection of 29 *S. aureus* isolates, seven other staphylococci, and three strains of other Gram-positive pathogens were used to determine the host range of LM12 and corresponding EOP (Table 1). Interestingly, LM12 has a broad spectrum of activity, showing a lytic effect in all *S. aureus* strains tested, infecting 22 out of the 29 *S. aureus* strains tested. Moreover, it shows a lytic effect against all other *Staphylococcus* spp. strains, excluding the *S. haemolyticus* strain tested. Furthermore, no lysis was observed on non-staphylococcal strains. In some cases, lysis from external events were observed, as phage haloes were observed with high phage concentrations, but no plaques could be seen when decreasing the phage titer. This phenomenon was mainly present on non-*S. aureus* staphylococcal strains.

Regarding the EOP experiments, in general, LM12 showed high to moderate EOP in the majority of the strains.

### 3.3. Antibacterial Assays Show That LM12 Can Inhibit S. aureus Biofilms

Phage infection was performed on Sa12 24-h biofilms, using a Multiplicity of Infection (MOI) of 1. According to the infection results, Sa12 can significantly decrease the number of viable biofilm cells about one order of magnitude during the first 6 h of infection (*p* < 0.01). However, after 24 h, the biofilm cells might have acquired resistance to the phage, and consequently, the numbers of cells after 24 h of infection increased (Figure S1).

### 3.4. LM12 Genome Is Similar to Kayvirus Genomes

The complete genomic sequence of bacteriophage LM12 was determined and deposited in GenBank under the accession number MG721208. LM12 has a linear dsDNA genome consisting of 143,652 bp, with a GC content of 29.3%. Semi-automatic annotation predicted that LM12 encodes 227 proteins, of which 70 have a putative function and 157 are considered hypothetical/novel (Table S1). LM12 genes are packed tightly, occupying ~90% of the genome. Most predicted gene products exhibit homology to known proteins of phages belonging to the *Kayvirus* genus, mostly to *Staphylococcus* phages G1, GH15, and P108. Moreover, three tRNA coding for Asp, Phe, and Trp were detected before the lytic module (gp157), and one tRNA coding for Met was detected between two genes with an unknown function (*gp197A* and *gp198*).

As in other phages of this genus, long-terminal repeats (LTRs) were detected at the genome ends. Although the exact borders between the LTRs and the rest of phage DNA has not been determined, the region between the *bofl* and *treA* genes was estimated to have 11.5 Kb, which is longer than other kayviruses [10].

**Figure 2:**
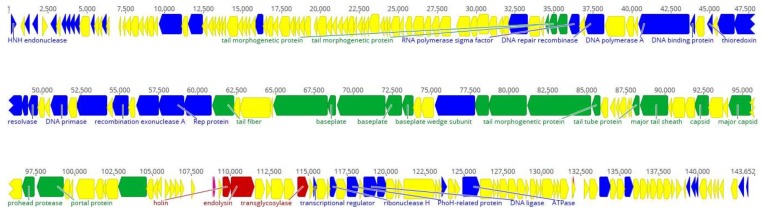
Genome overview of the *Staphylococcus* phage LM12. Genome map with predicted 227 coding sequences (CDSs) numbered and colored (yellow shows hypothetical proteins, blue shows DNA replication and transcription genes, green represents DNA packaging and phage morphogenesis genes, and red indicates cells lysis genes) according to their predicted function. Some important CDSs are highlighted. Above the genome, the nucleotide position in kb is given. The figure was performed using Geneious 9.1.4.

The LM12 genome is organized in functional modules, related to DNA replication and transcription, DNA packaging, phage morphogenesis, and cell lysis (Figure 2). On the left arm of LM12 genome are located genes encoding for DNA replication, recombination, and modification, namely nicotinamide phosphoribosyl transferase (gp25) and phosphoribosyl pyrophosphate synthetase (gp27); however, the majority of the genes found encode for proteins with unknown functions.

In the mid-range of the genome are located the majority of genes encoding for proteins related to DNA replication, recombination, and modification, including DNA polymerase A (gp89), ribonucleotide reductase (gp94 and gp95), DNA repair recombinase (gp85) and DNA primases/helicases (gp100, gp106, and gp108). Downstream in this region are found some structural genes, namely encoding the baseplate proteins (gp114, gp116, and gp117), tail-related proteins (gp115, gp 121–124, gp131–132), tail fiber proteins (gp109 and gp113), and capsid proteins (gp136 and gp139).

In the beginning of the right arm of the genome, genes encoding DNA packaging proteins, including portal protein (gp142) and terminase (gp149) are found, followed by the lytic module composed of a holin (gp158) and endolysin (gp159). Finally, a small group of DNA replication, recombination, and modification genes, including those encoding the ribonuclease H (gp169), PhoH-related protein (gp171), nucleoside 2-deoxyribosyltransferase (gp174), and serine/threonine protein phosphatase (gp200).

**Figure 3:**
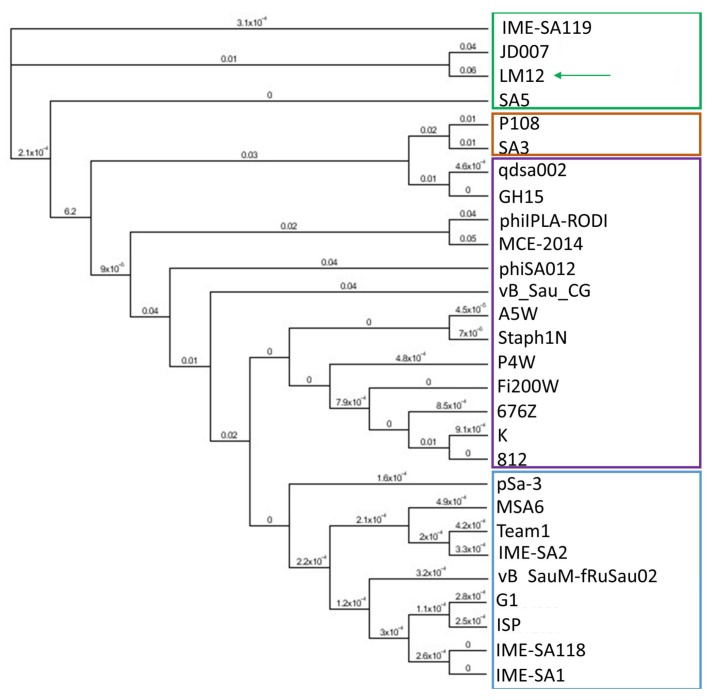
Phylogenetic tree from LM12 genome and its most closely related phage genomes. The tree was generated with a Maximum-Likelihood phylogenetic (PHYML) algorithm, with a bootstrapping of 100. Four groups are indicated (green, orange, purple, and blue), and arrows indicates phage LM12. The figure was performed using Geneious 9.1.4.

Moreover, 26 bacterial-origin promoters and 23 rho-independent terminators were detected. Whole-genome comparison analysis has shown that LM12 shares >90% identity and >90% coverage with the majority of the staphylococcal phages belonging to the *Kayvirus* genus. When the LM12 genome was aligned with 27 other kayviruses, the presence of four groups was visible on the generated tree (Figure 3). The group where phage LM12 was present is composed of three other phages, including IME-SA119 (KR908644), JD007 (NC_019726), and SA5 (JX875065), having 85%, 82%, and 83% identity, respectively. Moreover, LM12 shows identities <40% with the other 24 phages analyzed. Progressive Mauve analysis was performed between LM12, IME-SA119, JD007, and SA5 phages, in order to analyze sequence similarities. Results demonstrated that in general, LM12 is homologous with the other three phages, showing few noticeable differences (Figure S2). Several hypothetical proteins (including gp12–14, gp17–18, gp26, gp33, gp43, gp105, gp111, gp145, gp160, and gp198) are encoded within the more divergent genomic regions. Moreover, differences were observed in terminal repeat regions of TreA (gp15) and TreU (gp211). Comparing the four genomes, it was also possible to detect slight differences on the N-terminus of ribonucleotide reductase small subunit (gp94), and on the C-terminus of the tail morphogenetic protein (gp115).

### 3.5. LM12 Genome Encodes a Modular Lysin with Broad Bactericidal Activity

In silico analysis of LM12 identified gp159 as the endolysin (E-LM12). This protein has a molecular weight of about 57.74 KDa and a pI of 10.08. This is a 496 amino acid modular protein composed of a N-terminal **c**ysteine, **h**istidine-dependent **a**midohydrolase/**p**eptidase (CHAP) domain (PF05257), an Amidase-2 (PF01510) domain in the middle, and a SRC Homology 3 (SH3) (PF08460) cell-wall-binding domain on the C-terminus.

BLAST analysis revealed that E-LM12 shows high similarity with the endolysins from several staphylococcal phages, including *Staphylococcus* phage phiIPLA-RODI (100% coverage, 98% identity).

After expression in *E. coli*, the soluble fraction of E-LM12 was purified by nickel affinity chromatography, via its C-terminal 6× His-tag. Our procedure led to a purified stock concentration of 120–140 mg/L of protein in the induced *E. coli* culture. As expected, E-LM12 was active against a wide range of staphylococci strains, including MRSA clinical isolates (Table 1). Moreover, the lysin was unable to lyse other Gram-positive bacteria, such as *L. monocytogenes*, *E. faecium*, and *E. faecalis.*

E-LM12 activity was determined by an MIC assay. The endolysin was able to inhibit *S. aureus* Sa12 growth at concentrations of 34.5 ± 10.5 μg·mL^−1^.

### 3.6. E-LM12 Has an Anti-Biofilm Effect, Being Able to Lyse Exponential and Stationary Phase Cells

**Figure 4:**
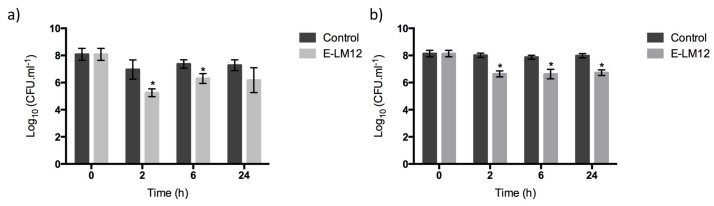
Endolysin E-LM12 activity against *S. aureus* biofilms, using 8 μM (**a**) intact biofilms; (**b**) scraped biofilms. Data was assessed by CFU counting, and the values represent the mean (plus or minus) of three independent experiments performed in duplicate. Statistical differences (*p* < 0.01) between the control biofilms and the LM12-treated biofilms (*) were determined by two-way repeated-measures analysis of variance (ANOVA) with a Sidak post-test.

The anti-biofilm potential of the endolysin E-LM12 was assessed (Figure 4a). Two hours after endolysin addition, the number of viable biofilm cells was reduced by about 1.7 orders of magnitude (*p* < 0.01). The reduction decreased slightly after 6 h, when a reduction of about 1.1 orders of magnitude was detected (*p* < 0.01). This reduction was maintained after 24 h of infection, which suggests that no endolysin resistance was acquired by biofilm cells.

The influence of the biofilm’s 3D structure on lysin activity was assessed by scraping the biofilm, in order to disturb its complex 3D structure (Figure 4b). Results have shown that E-LM12 reduced the number of viable cells by about 1.3 orders of magnitude in all time points tested (*p* < 0.01), suggesting that there are no significant differences on endolysin efficacy against intact or physically disturbed biofilms.

To get a deeper understanding of endolysin–cell interactions, the protein efficacy was tested against exponential, stationary, and biofilm cells.

**Figure 5:**
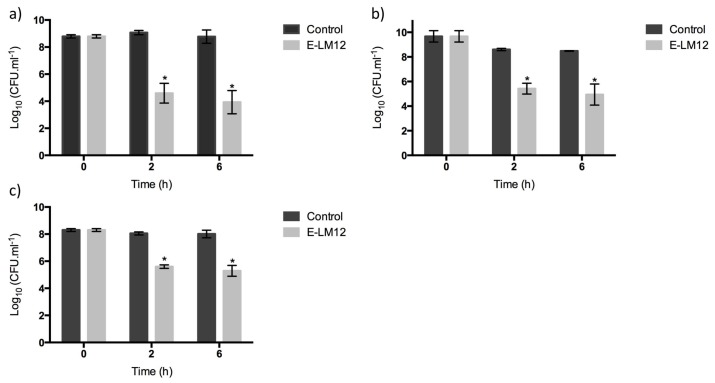
Endolysin E-LM12 activity against 24 h *S. aureus* planktonic cells, using 8 μM (**a**) exponential phase cells, (**b**) stationary phase cells, and (**c**) biofilm suspended cells. Data was assessed by CFU counting, and the values represent the mean (plus or minus) of three independent experiments performed in duplicate. Statistical differences (*p* < 0.01) between control biofilms and LM12-treated biofilms (*) were determined by two-way repeated-measures analysis of variance (ANOVA) with a Sidak post-test.

E-LM12 demonstrated a high bactericidal activity against exponential-phase cells, reducing the number of viable cells by approximately 4.5 and 4.8 orders of magnitude after 2 h and 6 h, respectively (*p* < 0.01) (Figure 5a). Moreover, this endolysin was very active against stationary-phase cells, as it was able to reduce the cell numbers by 3.2 orders of magnitude, which was maintained for six hours (*p* < 0.01) (Figure 5b).

To understand the influence of the biofilm matrix in lysin efficacy, biofilms were disrupted and collected into a homogeneous suspension of biofilm cells (Figure 5c). E-LM12 was active against biofilm cells, reducing them by 2.7 orders of magnitude in the first 2 h, which was slightly increased to three orders of magnitude after 6 h of infection (*p* < 0.01).

## 4. Discussion

Nowadays, the emergence of multidrug-resistant strains is a main concern [33]. In fact, the WHO recently suggested that the scientific community and pharma industries develop new antimicrobials to combat multi-resistant pathogens, namely the MRSA, VISA, and VRSA strains [1]. The use of lytic bacteriophages and their cell-wall-degrading enzymes (endolysins) have been catching the attention of both researchers and industry as possible tools to combat AMR pathogens. To achieve that, we aimed to isolate phages with lytic activity against *S. aureus*.

As a general strategy, we searched for lytic bacteriophages from environmental sewage waters, using a set of different clinical MRSA isolates to be combined in the sewage enrichment. When Paradela (Vila Verde) effluents were used, it was possible to isolate a *S. aureus* phage. The selected phage was analyzed by TEM, and images suggested that it belongs to the *Kayvirus* genus [34], formerly known as *Twortlikevirus* [35]. The phage was named vB_SauM_LM12, according to Kropinski et al.’s [36] recommendations. After sequencing, LM12 was included in the Class III group, as defined by Kwan [37], due to its myovirus morphology, genome size between 140–150 Kbp, and proper gene organization. General genome features—such as G + C content, number of genes, tRNA genes, and genome organization—related LM12 with phage K, which is one of the well-studied *S. aureus* strictly lytic phages [38]. Comparative genomics studies have suggested that LM12 belongs to the *Kayvirus* genus, clustering with members of the *Staphylococcus* virus JD7 species. The modular feature of the phage genome is stressed by the presence of *S. aureus* σ70 promoters and rho-independent terminators. One particularity of the LM12 genome is the absence of introns, which are very common among *Staphylococcus* myoviruses [10]. A similar phenomenon was described for *Staphylococcus* phage GH15, where no introns were detected in its genes for encoding critical enzymatic functions [39]. Recently, Abatangelo et al. [40], suggested that this event might indicate intron loss. Overall, the high genomic similarities and the gene organization suggest genome mosaicism among staphylococcal myoviruses, which is in accordance with the model of modular evolution among phages. In this model, horizontal gene transfer events may lead to new genetic combinations [41].

Usually, *Staphylococcus* myoviruses are polyvalent phages with strictly virulent behavior, displaying a broad host range. For those reasons, they are considered the most promising phages from a therapeutic point of view [35]. The lytic potential of Kayviruses against *S. aureus* has been shown in different settings [11,12,13,42]. LM12 was no exception, as it showed a polyvalent behavior lysing all *S. aureus* tested strains, regardless their origin. Usually, viruses of this family adsorb to staphylococcal cells, using the anionic backbone of the cell wall teichoic acid (WTA) as a receptor [43]. More recently, it was shown that myovirus ΦSA012 possesses at least two receptor binding proteins (RBP), one binding to the α-GlcNAc in WTAs and the other expected to bind the WTA backbone [44]. Both ΦSA012 proteins have homologous proteins in LM12 genome, namely gp109 and gp111. The presence of more than one RBP can justify the broad spectra of Kayviruses.

As more than 60% of all infections are caused by biofilms [45], the anti-biofilm potential of LM12 was tested. Although several studies focus on biofilm biomass reduction [46,47], the purpose of this study was to assess LM12’s direct effect on biofilm cells. During the first 6 h of infection, 90% of the biofilm cells were killed. However, a regrowth was observed at 24 h of infection, which might be related with the emergence of phage-resistant mutants, something already observed when biofilms are challenged by a single phage [48]. Even so, given the broad lytic spectra of LM12 and its quick anti-biofilm potential, the inclusion of LM12 in a cocktail with phages with complementary host ranges could constitute a valuable weapon against AMR *Staphylococcus*, and prevent the emergence of phage-resistant phenotypes [49].

Besides phages, phage-derived endolysins have been widely studied against *S. aureus*. Although some endolysins are effective against *S. aureus* biofilms [50,51,52], few studies are detailed in lysin–biofilm interactions. The endolysin from phage LM12 was cloned, heterologously expressed, and purified. The purified enzyme was named E-LM12, and showed activity against all *S. aureus* strains tested, namely MRSA clinical isolates. Curiously, no effect was observed against non-*S. aureus* staphylococci. The amino acid sequence of E-LM12 is very conserved, being closely related to several staphylococcal endolysins [53]. Therefore, it was not surprising that the MIC of E-LM12 was comparable to previous studies with other lysins, namely LysK [30,54]. In addition, it was demonstrated that E-LM12 could disrupt biofilms, causing greater than 90% reduction of the biofilm cells, at least up to 24 h. In opposition to the phage killing assays, no increase on the biofilm biomass was detected at 24 h of infection, suggesting that there were no resistance mechanisms developed by the biofilm cells against the endolysins. This absence of resistance to phage’s endolysins is in accordance to what has been described [55].

Although the reduction obtained on biofilms was significant, it was considered relevant to understand the influence of the physiological state of the cells with E-LM12 activity, as well as the influence of the complex 3D structure of the biofilm. As biofilms are generally created by bacterial cells that are in a wide range of physiological states, E-LM12 was tested against exponential- and late stationary-phase cells. Although the endolysin E-LM12 has shown to be highly active against both types of cells, higher reductions were observed in exponential-phase cells. *S. aureus* stationary-phase cells have been shown to have thicker cell walls, with non-uniformly thickened septa, in comparison to exponential-phase cells [56]. Furthermore, the fact that around 12% of the stems in the peptidoglycan do not have pentaglycine bridges attached [56] might justify the lower activity of the studied endolysin on stationary-phase cells, as the CHAP domain cleaves interpeptide bridges exclusively [57], and the SH3 binding domain recognizes pentaglycine cross bridges in the peptidoglycan [58]. The fact that E-LM12 could infect biofilm cells within the same orders of magnitude as stationary-phase cells suggested that lysin efficacy was not highly affected by cell physiology. A similar phenomenon was detected in the artificially-modified lysin, Art-240, against streptococci [59].

The biofilm matrix can act as a barrier, per se, conferring protection to the biofilm cells—for example, against antibiotics [8]. As the studied endolysin was active against cells with different morphological/physiological states, E-LM12 was tested against biofilms whose architecture was mechanically disrupted (scraped biofilms). Surprisingly, the endolysin activity was not improved by this light disruption, suggesting that the reminiscent cellular aggregates are still protected by the biofilm matrix, and that this matrix interferes with LM12 activity.

Taken together, phage LM12 and its endolysin seem to have a strong and broad host range effect against *S. aureus*. Their anti-biofilm potential could be increased by using a combination of different phages and endolysins [60], or by using them in combination with matrix-disrupting chemicals [61]. In conclusion, phage LM12, and mainly its endolysin E-LM12, present strong potential in the development of new products to control the highly pathogenic and antibiotic-resistant *Staphylococcus aureus*.

## Figures and Tables

**Table 1:** Phage vB_SauM-LM12 (LM12) spectrum of activity with efficiency of plating (EOP) and E-LM12 lytic spectrum. High, moderate, and low efficiency of plating represent >10%, 0.01–9%, and <0.01%, respectively.

Species	Strain	Origin	Antibiotic Resistance	Phage Infectivity	EOP	Endolysin Activity
*S. aureus*	ATCC 25923	Clinical Isolate	Unknown	+	High	+
	ATCC BAA-976	Tracheal aspirate	Unknown	+	Medium	−
	CECT 239	Human lesion	Unknown	+	High	+
	Sa1	Expectoration	MRSA	+	Medium	+
	Sa2	Expectoration	MRSA	+	Low	+
	Sa3	Expectoration	MRSA	LFW ^1^	N/A ^2^	+
	Sa4	Pus	MRSA	+	Medium	+
	Sa5	Bronchial aspirate	MRSA	+	High	+
	Sa6	Expectoration	MRSA	LFW	N/A	+
	Sa7	Expectoration	MRSA	+	High	+
	Sa8	Expectoration	MRSA	+	High	+
	Sa9	Urine	MRSA	LFW	N/A	+
	Sa10	Skin exudate	MRSA	+	High	+
	Sa11	Skin exudate	MRSA	+	Medium	+
	Sa12	Urine	MRSA	+	High	+
	Sa13	Skin exudate	MRSA	+	Medium	+
	Sa14	Urine	MRSA	LFW	N/A	+
	Sa15	Expectoration	MRSA	+	High	+
	Sa16	Bronchial aspirate	MRSA	+	Medium	+
	Sa17	Nasal exudate	MRSA	+	LFW	+
	Sa18	Expectoration	MRSA	LFW	N/A	+
	Sa19	Expectoration	MRSA	+	High	+
	Sa20	Urine	MRSA	+	Medium	+
	Sa21	Urine	MRSA	+	High	+
	Sa22	Expectoration	MRSA	+	High	+
	Sa23	Expectoration	MRSA	+	High	+
	Sa24	Skin exudate	MRSA	LFW	N/A	+
	Sa25	Expectoration	MRSA	+	High	+
	Sa26	Expectoration	MRSA	LFW	N/A	+
*S. epidermidis*	RP62A	Catheter-associated sepsis	Unknown	LFW	N/A	−
	9142	Blood culture	Unknown	+	Low	−
*S. haemolyticus*	SECOM 065A.1 [15]	Healthy Skin	Unknown	−	−	−
*S. equorum*	SECOM060A [15]	Healthy Skin	Unknown	LFW	N/A	−
*S. capitis*	SECOM052A [15]	Healthy Skin	Unknown	+	High	−
*S. warneri*	SECOMF16 [15]	Healthy Skin	Unknown	+	Medium	−
*S. hominis*	SECOMM11 [15]	Healthy Skin	Unknown	LFW	N/A	−
*Enterococcus faecalis*	CECT 184	Milk	Unknown	−	−	−
*Enterococcus faecium*	CECT 410	Unknown	Unknown	−	−	−
*Listeria monocytogenes*	CECT 5725	Chicken	Unknown	−	−	−

^1^ LFW: Lysis from without; ^2^ N/A—non available.

## Supplementary Materials

The following are available online at http://www.mdpi.com/1999-4915/10/4/182/s1, Table S1: Features of LM12 predicted coding sequences (CDSs). For each CDS, the transcription start and stop position and the coding strand is given. At protein level, the corresponding gene product size, molecular weight, and pI, as well as the homolog predicted function, homologs, and E-values and motifs are shown; Figure S1: Phage LM12 infection on 24 h *S. aureus* biofilms, using a multiplicity of infection (MOI) 1. Data was assessed by colony forming units (CFU) counting, and the values represent the mean (plus or minus) of three independent experiments performed in duplicate. Statistical differences (*p* < 0.01) between control biofilms and LM12-treated biofilms (*) were determined by two-way repeated-measures analysis of variance (ANOVA) with a Sidak post-test; Figure S2: Multiple genome alignment of *Staphylococcus* phage LM12 with closest homologs. The figure was performed using Geneious 9.1.4.
