# Supplementary material for: Characterization of a New Staphylococcus aureus Kayvirus Harboring a Lysin Active against Biofilms
Source: Viruses. 2018 Apr 7;10(4):182. doi: 10.3390/v10040182 (PMC5923476; doi:10.3390/v10040182)
Supplement: Supplementary file 1 [file viruses-10-00182-s001.pdf]

**Table S1.** Features of LM12 predicted coding sequences (CDSs). For each CDS the transcription start and stop position, and the coding strand is given. At protein level the corresponding gene product size molecular weight and pI as well as the homolog predicted function, homology values and motifs are shown

| DNA | | | | Protein | | | | | | |
|---|---|---|---|---|---|---|---|---|---|---|
| **CDS** | **Coordinates** | **Strand** | **Length** | **Mass** | **pI** | **aa residues** | **Function** | **Homolog** | **E-value** | **Motif** |
| 1 | 156..473 | + | 318 | 12.70 | 9.99 | 105 | HNH endonuclease | ORF085 [Staphylococcus virus G1] | 6,00E-71 | HNH_3 (PF13392) HNH endonuclease; HTH_23 (PF13384) Homeodomain-like domain |
| 2 | 790..981 | - | 192 | 7.67 | 10.61 | 63 | hypothetical protein | ORF221 [Staphylococcus virus G1] | 6,00E-37 | |
| 3 | 1,031..1,318 | - | 288 | 11.06 | 6.52 | 95 | TreK | hypothetical protein [Staphylococcus phage [illegible] | 1,00E-61 | |
| 4 | 1,524..1,832 | - | 309 | 11.80 | 4.57 | 102 | TreJ | TreJ [Staphylococcus phage phiIPLA-RODI] | 1,00E-69 | |
| 5 | 2,143..2,481 | + | 339 | 13.47 | 4.25 | 112 | hypothetical protein | hypothetical protein [Staphylococcus phage phiIPLA-RODI] | 9,00E-72 | |
| 6 | 2,686..3,033 | - | 348 | 13.66 | 4.26 | 115 | TreH | hypothetical protein [Staphylococcus phage [illegible] | 4,00E-77 | |
| 7 | 3,044..3,283 | - | 240 | 9.18 | 6.07 | 79 | hypothetical protein | hypothetical protein CPT_phageK_gp007 [Staphylococcus virus K] | 4,00E-50 | |
| 8 | 3,376..3,633 | - | 258 | 10.24 | 6.52 | 85 | TreF | ORF175 [Staphylococcus virus G1] | 5,00E-55 | |
| 9 | 3,637..3,93 | - | 294 | 11.59 | 4.09 | 97 | TreE | TreE [Staphylococcus phage vB_Sau_CG] | 7,00E-60 | |
| 10 | 3,93..4,217 | - | 288 | 10.90 | 7.85 | 95 | TreD | ORF158 [Staphylococcus virus G1] | 8,00E-61 | |
| 11 | 4,217..4,507 | - | 291 | 11.30 | 4.03 | 96 | TreC | terminal repeat-encoded protein [Staphylococcus phage P108] | 5,00E-60 | |
| 12 | 4,536..5,171 | - | 636 | 24.80 | 9.77 | 211 | hypothetical protein | hypothetical protein [Staphylococcus phage SA3] | 4,00E-66 | |
| 0012A | 5,269..5,46 | - | 192 | 7.19 | 6.51 | 63 | TreB | TreB [Staphylococcus phage vB_Sau_S24] | 2,00E-26 | |
| 13 | 5,467..5,769 | - | 303 | 11.22 | 5.10 | 100 | hypothetical protein | hypothetical protein vBSauClo6_132 [Staphylococcus phage vB_Sau_Clo6] | 2,00E-61 | |
| 14 | 5,772..5,954 | - | 183 | 6.50 | 8.49 | 60 | hypothetical membrane protein | hypothetical protein [Staphylococcus phage S25-[illegible] | 5,00E-15 | |
| 15 | 5,951..6,244 | - | 294 | 11.18 | 4.12 | 97 | TreA | hypothetical protein [Staphylococcus phage MCE-2014] | 3,00E-58 | |
| 16 | 6,26..6,43 | - | 171 | 6.27 | 4.26 | 56 | hypothetical membrane protein | hypothetical protein P108_0115 [Staphylococcus phage P108] | 3,00E-28 | |
| 17 | 7,061..7,174 | - | 114 | 4.27 | 3.86 | 37 | hypothetical protein | | | |

| | | | | | | | | | | |
|---|---|---|---|---|---|---|---|---|---|---|
| 18 | 7,314..7,463 | - | 150 | 5.88 | 4.21 | 49 | hypothetical protein | hypothetical protein [Staphylococcus phage phiIPLA-C1C] | 2,00E-07 | |
| 19 | 7,463..7,798 | - | 336 | 12.88 | 4.84 | 111 | hypothetical protein | ORF131 [Staphylococcus virus G1] | 3,00E-72 | |
| 20 | 7,8..8,309 | - | 510 | 20.32 | 9.69 | 169 | hypothetical protein | hypothetical protein [Staphylococcus phage MCE-2014] | 2,00E-116 | |
| 21 | 8,312..8,623 | - | 312 | 11.61 | 4.91 | 103 | hypothetical protein | ORF142 [Staphylococcus virus G1] | 1,00E-66 | |
| 22 | 8,689..8,886 | - | 198 | 7.86 | 4.11 | 65 | hypothetical protein | hypothetical protein [Staphylococcus phage MCE-2014] | 2,00E-39 | |
| 23 | 8,888..9,28 | - | 393 | 15.44 | 4.79 | 130 | hypothetical protein | ORF113 [Staphylococcus virus G1] | 2,00E-87 | |
| 24 | 9,3..9,545 | - | 246 | 9.96 | 9.23 | 81 | hypothetical protein | ORF178 [Staphylococcus virus G1] | 4,00E-52 | |
| 25 | 9,624..11,093 | - | 1470 | 56.14 | 5.05 | 489 | nicotinamide phosphoribosyl transferase | putative nicotinamide phosphoribosyl transferase [Staphylococcus phage GH15] | 0,00E+00 | NAPRTase (PF04095) Nicotinate phosphoribosyltransferase (NAPRTase) family |
| 26 | 11,097..11,492 | - | 396 | 15.41 | 5.06 | 131 | hypothetical protein | | | |
| 27 | 11,507..12,415 | - | 909 | 35.02 | 4.74 | 302 | phosphoribosyl pyrophosphate synthetase | phosphoribosyl pyrophosphate synthetase [Staphylococcus phage phiIPLA-RODI] | 0,00E+00 | |
| 28 | 12,412..12,705 | - | 294 | 11.42 | 6.54 | 97 | hypothetical protein | hypothetical protein GH15_201 [Staphylococcus phage GH15] | 1,00E-64 | |
| 29 | 12,798..13,088 | - | 291 | 11.23 | 9.71 | 96 | hypothetical membrane protein | putative membrane protein [Staphylococcus phage GH15] | 1,00E-58 | |
| 30 | 13,089..13,289 | - | 201 | 7.60 | 4.88 | 66 | hypothetical protein | ORF211 [Staphylococcus virus G1] | 8,00E-42 | |
| 31 | 13,302..13,526 | - | 225 | 8.50 | 6.54 | 74 | hypothetical protein | hypothetical protein [Staphylococcus phage MCE-2014] | 2,00E-45 | |
| 32 | 13,542..13,7 | - | 159 | 5.78 | 4.45 | 52 | hypothetical membrane protein | hypothetical protein [Staphylococcus phage MCE-2014] | 1,00E-21 | |
| 33 | 13,716..13,919 | - | 204 | 7.74 | 5.10 | 67 | hypothetical protein | hypothetical protein GH15_196 [Staphylococcus phage GH15] | 4,00E-38 | |
| 34 | 13,997..14,677 | - | 681 | 25.89 | 4.80 | 226 | hypothetical protein | hypothetical protein [Staphylococcus phage [illegible] | 1,00E-109 | |
| 35 | 14,678..14,995 | - | 318 | 12.08 | 4.54 | 105 | hypothetical protein | hypothetical protein GH15_194 [Staphylococcus phage GH15] | 2,00E-65 | |
| 36 | 14,998..15,261 | - | 264 | 10.35 | 4.50 | 87 | hypothetical protein | hypothetical protein GH15_193 [Staphylococcus phage GH15] | 1,00E-51 | |
| 37 | 15,276..15,455 | - | 180 | 6.39 | 10.79 | 59 | hypothetical membrane protein | hypothetical protein GH15_192 [Staphylococcus phage GH15] | 1,00E-28 | |

| | | | | | | | | | | |
|---|---|---|---|---|---|---|---|---|---|---|
| 38 | 15,501..15,812 | - | 312 | 12.51 | 10.49 | 103 | hypothetical protein | hypothetical protein P108_0092 [Staphylococcus phage P108] | 2,00E-67 | |
| 39 | 15,793..16,32 | - | 528 | 20.62 | 6.87 | 175 | metallophosphatase | hypothetical protein GH15_189 [Staphylococcus phage GH15] | 4,00E-123 | |
| 40 | 16,317..16,553 | - | 237 | 9.16 | 8.39 | 78 | hypothetical protein | hypothetical protein GH15_188 [Staphylococcus phage GH15] | 7,00E-49 | |
| 41 | 16,558..16,962 | - | 405 | 15.24 | 9.63 | 134 | hypothetical membrane protein | hypothetical protein [Staphylococcus phage [illegible] | 3,00E-73 | |
| 42 | 16,962..17,267 | - | 306 | 11.62 | 4.68 | 101 | hypothetical protein | hypothetical protein [Staphylococcus phage pSco-[illegible] | 2,00E-56 | |
| 43 | 17,344..18,009 | - | 666 | 24.89 | 4.43 | 221 | hypothetical protein | hypothetical protein vBSauCG_109 [Staphylococcus phage vB_Sau_CG] | 7,00E-146 | |
| 44 | 18,025..18,297 | - | 273 | 10.54 | 10.65 | 90 | hypothetical protein | hypothetical protein P108_0087 [Staphylococcus phage P108] | 5,00E-53 | |
| 45 | 18,29..18,403 | - | 114 | 4.47 | 4.44 | 37 | hypothetical protein | hypothetical protein GH15_184 [Staphylococcus phage GH15] | 9,00E-15 | bZIP_1 (PF00170) BZIP domain |
| 46 | 18,414..18,641 | - | 228 | 8.26 | 4.83 | 75 | hypothetical membrane protein | hypothetical protein [Staphylococcus phage pSco-[illegible] | 7,00E-44 | |
| 47 | 18,657..18,95 | - | 294 | 11.65 | 3.76 | 97 | hypothetical protein | hypothetical protein [Staphylococcus phage [illegible] | 3,00E-62 | |
| 48 | 18,952..19,362 | - | 411 | 15.46 | 4.36 | 136 | hypothetical membrane protein | membrane protein [Staphylococcus phage phiIPLA-RODI] | 5,00E-90 | |
| 49 | 19,588..19,767 | - | 180 | 7.25 | 7.35 | 59 | hypothetical protein | hypothetical protein GH15_180 [Staphylococcus phage GH15] | 2,00E-35 | |
| 50 | 19,781..20,383 | - | 603 | 23.36 | 10.38 | 200 | hypothetical protein | hypothetical protein GH15_179 [Staphylococcus phage GH15] | 3,00E-145 | |
| 51 | 20,383..20,733 | - | 351 | 13.68 | 10.52 | 116 | hypothetical protein | hypothetical protein GH15_178 [Staphylococcus phage GH15] | 4,00E-77 | |
| 52 | 20,748..21,053 | - | 306 | 12.14 | 9.71 | 101 | hypothetical protein | hypothetical protein GH15_177 [Staphylococcus phage GH15] | 6,00E-68 | |
| 53 | 21,123..21,395 | - | 273 | 10.00 | 4.11 | 90 | hypothetical membrane protein | membrane protein [Staphylococcus phage phiIPLA-RODI] | 9,00E-55 | |
| 54 | 21,401..21,748 | - | 348 | 12.97 | 4.32 | 115 | hypothetical protein | hypothetical protein [Staphylococcus phage MCE-2014] | 1,00E-77 | |
| 55 | 21,761..22,129 | - | 369 | 14.16 | 4.03 | 122 | hypothetical protein | hypothetical protein [Staphylococcus phage MCE-2014] | 3,00E-83 | |
| 56 | 22,142..22,324 | - | 183 | 7.14 | 9.03 | 60 | hypothetical membrane protein | hypothetical protein P108_0075 [Staphylococcus phage P108] | 1,00E-33 | |
| 57 | 22,372..22,668 | - | 297 | 11.47 | 8.82 | 98 | hypothetical protein | hypothetical protein [Staphylococcus phage phiIPLA-RODI] | 5,00E-64 | |

| | | | | | | | | | |
|---|---|---|---|---|---|---|---|---|---|
| 58 | 22,661..22,837 | - | 177 | 7.01 | 10.67 | 58 | hypothetical protein | hypothetical protein [Staphylococcus phage MCE-2014] | 2,00E-31 |
| 59 | 22,849..23,097 | - | 249 | 9.05 | 4.50 | 82 | hypothetical membrane protein | hypothetical protein P108_0072 [Staphylococcus phage P108] | 1,00E-45 |
| 60 | 23,113..23,757 | - | 645 | 25.10 | 6.06 | 214 | hypothetical protein | hypothetical protein [Staphylococcus phage MCE-2014] | 6,00E-148 |
| 61 | 23,838..24,071 | - | 234 | 8.93 | 9.00 | 77 | hypothetical protein | hypothetical protein P108_0070 [Staphylococcus phage P108] | 5,00E-48 |
| 62 | 24,064..24,312 | - | 249 | 9.09 | 9.66 | 82 | hypothetical membrane protein | hypothetical protein [Staphylococcus phage [illegible] | 1,00E-49 |
| 63 | 24,305..24,481 | - | 177 | 6.99 | 4.26 | 58 | hypothetical protein | ORF240 [Staphylococcus virus G1] | 4,00E-33 |
| 64 | 24,517..25,074 | - | 558 | 21.68 | 10.07 | 185 | hypothetical membrane protein | membrane protein [Staphylococcus phage phiIPLA-RODI] | 6,00E-132 |
| 65 | 25,079..25,321 | - | 243 | 9.39 | 9.94 | 80 | hypothetical protein | ORF182 [Staphylococcus virus G1] | 5,00E-49 |
| 66 | 25,468..25,866 | - | 399 | 15.45 | 9.53 | 132 | hypothetical membrane protein | putative membrane protein [Staphylococcus phage GH15] | 2,00E-90 |
| 67 | 25,929..26,633 | - | 705 | 27.46 | 4.33 | 234 | hypothetical protein | hypothetical protein [Staphylococcus phage MCE-2014] | 4,00E-159 |
| 68 | 26,65..27,093 | - | 444 | 17.54 | 6.29 | 147 | hypothetical protein | hypothetical protein GH15_162 [Staphylococcus phage GH15] | 2,00E-99 |
| 69 | 27,158..27,616 | - | 459 | 17.85 | 4.58 | 152 | hypothetical protein | hypothetical protein GH15_161 [Staphylococcus phage GH15] | 2,00E-106 |
| 70 | 27,634..28,365 | - | 732 | 28.36 | 5.09 | 243 | hypothetical protein | hypothetical protein qdsa002_176 [Staphylococcus phage qdsa002] | 3,00E-173 |
| 71 | 28,737..29,6 | - | 864 | 32.33 | 5.47 | 287 | hypothetical protein | ORF036 [Staphylococcus virus G1] | 0,00E+00 |
| 72 | 29,6..30,046 | - | 447 | 17.34 | 10.90 | 148 | hypothetical protein | ORF099 [Staphylococcus virus G1] | 3,00E-104 |
| 73 | 30,024..30,791 | - | 768 | 30.05 | 10.00 | 255 | hypothetical protein | hypothetical protein [Staphylococcus phage [illegible] | 0,00E+00 |
| 74 | 30,784..31,32 | - | 537 | 20.83 | 7.71 | 178 | hypothetical protein | ORF075 [Staphylococcus virus G1] | 4,00E-128 |
| 75 | 31,384..31,695 | - | 312 | 12.01 | 4.41 | 103 | hypothetical protein | ORF143 [Staphylococcus virus G1] | 7,00E-69 |
| 76 | 31,682..32,05 | - | 369 | 14.00 | 5.75 | 122 | hypothetical membrane protein | ORF118 [Staphylococcus virus G1] | 2,00E-81 |
| 77 | 32,064..33,314 | - | 1251 | 47.57 | 6.01 | 416 | metallophosphoesterase | DNA polymerase [Staphylococcus phage phiIPLA-RODI] | 0,00E+00 |
| 78 | 33,307..34,062 | - | 756 | 29.16 | 4.19 | 251 | hypothetical protein | ORF046 [Staphylococcus virus G1] | 0,00E+00 |
| 79 | 34,066..34,326 | - | 261 | 10.29 | 5.71 | 86 | hypothetical protein | ORF174 [Staphylococcus virus G1] | 2,00E-55 |
| 80 | 34,422..34,649 | - | 228 | 7.82 | 4.12 | 75 | tai morphogenetic protein | ORF189 [Staphylococcus virus G1] | 1,00E-45 |

| | | | | | | | | | | |
|---|---|---|---|---|---|---|---|---|---|---|
| 81 | 34,664..35,176 | - | 513 | 17.85 | 4.09 | 170 | tai morphogenetic protein | putative bacterial adhesin/Ig-like protein [Staphylococcus virus K] | 6,00E-115 | Big_2 (PF02368) Bacterial Ig-like domain (group 2) |
| 82 | 35,199..35,831 | - | 633 | 23.20 | 4.44 | 210 | putative Ig-like protein | ORF059 [Staphylococcus virus G1] | 1,00E-150 | |
| 83 | 35,959..36,621 | - | 663 | 26.61 | 5.02 | 220 | RNA polymerase sigma factor | ORF056 [Staphylococcus virus G1] | 5,00E-157 | |
| 84 | 36,608..36,961 | - | 354 | 13.38 | 4.83 | 117 | hypothetical protein | ORF121 [Staphylococcus virus G1] | 6,00E-80 | |
| 85 | 36,965..38,221 | - | 1257 | 46.77 | 4.89 | 418 | DNA repair recombinase | ORF021 [Staphylococcus virus G1] | 0,00E+00 | RecA (PF00154) recA bacterial DNA recombination protein; Rad51 (PF08423) Rad51; ATPase (PF06745) KaiC |
| 86 | 38,281..39,552 | - | 1272 | 46.90 | 4.37 | 423 | hypothetical protein | hypothetical protein [Staphylococcus phage S25-[illegible] | 0,00E+00 | |
| 87 | 39,639..40,121 | - | 483 | 18.95 | 5.32 | 160 | hypothetical protein | ORF089 [Staphylococcus virus G1] | 9,00E-116 | |
| 88 | 40,138..40,38 | - | 243 | 9.09 | 3.82 | 80 | hypothetical protein | hypothetical protein GH15_142 [Staphylococcus phage GH15] | 2,00E-50 | |
| 89 | 40,449..43,667 | - | 3219 | 124.62 | 5.11 | 1072 | DNA polymerase A | DNA polymerase I [Staphylococcus phage phiSA12] | 0,00E+00 | DNA_pol_A (PF00476) DNA polymerase family A; DNA_pol_A_exo1 (PF01612) 3'-5' exonuclease |
| 90 | 43,743..44,048 | - | 306 | 11.93 | 5.83 | 101 | DNA binding protein | ORF147 [Staphylococcus virus G1] | 5,00E-67 | Bac_DNA_binding (PF00216) Bacterial DNA binding protein |
| 91 | 44,058..44,618 | - | 561 | 22.11 | 6.36 | 186 | hypothetical protein | hypothetical protein GH15_139 [Staphylococcus phage GH15] | 8,00E-133 | |
| 92 | 44,861..45,181 | - | 321 | 12.05 | 4.54 | 106 | thioredoxin-like protein | oxidoreductase [Staphylococcus phage MCE-2014] | 1,00E-69 | Thioredoxin (PF00085) Thioredoxin |
| 93 | 45,165..45,494 | - | 330 | 12.46 | 4.36 | 109 | hypothetical protein | hypothetical protein [Staphylococcus phage MCE-2014] | 2,00E-72 | |
| 94 | 45,512..46,561 | - | 1050 | 40.49 | 4.43 | 349 | ribonucleotide reductase small subunit | ribonucleotide reductase small subunit [Staphylococcus phage phiIPLA-RODI] | 0,00E+00 | Ribonuc_red_sm (PF00268) Ribonucleotide reductase, small chain |

| | | | | | | | | | | |
|---|---|---|---|---|---|---|---|---|---|---|
| 95 | 46,575..48,689 | - | 2115 | 80.32 | 5.41 | 704 | ribonucleotide reductase large subunit | ribonucleotide reductase large subunit [Staphylococcus phage phiIPLA-RODI] | 0,00E+00 | Ribonuc_red_lgC (PF02867) Ribonucleotide reductase, barrel domain; RNR_N (PF08343) Ribonucleotide reductase N-terminal; Ribonuc_red_lgN (PF00317) Ribonucleotide reductase, all-alpha domain |
| 96 | 48,704..49,096 | - | 393 | 14.75 | 10.45 | 130 | ribonucleotide reductase stimulatory protein | putative NrdI protein [Staphylococcus phage GH15 | 6,00E-89 | Flavodoxin_NdrI (PF07972) NrdI Flavodoxin like |
| 97 | 49,114..49,722 | - | 609 | 23.62 | 5.15 | 202 | resolvase | hypothetical protein P108_0033 [Staphylococcus phage P108] | 2,00E-147 | |
| 98 | 49,709..50,161 | - | 453 | 17.05 | 4.52 | 150 | hypothetical protein | hypothetical protein P108_0032 [Staphylococcus phage P108] | 6,00E-101 | |
| 99 | 50,161..50,499 | - | 339 | 12.94 | 3.87 | 112 | hypothetical protein | ORF127 [Staphylococcus virus G1] | 1,00E-71 | |
| 100 | 50,565..51,632 | - | 1068 | 40.95 | 8.58 | 355 | DNA primase | DNA primase [Staphylococcus phage JD007] | 0,00E+00 | zf-CHC2 (PF01807) CHC2 zinc finger |
| 101 | 51,647..52,243 | - | 597 | 23.24 | 6.81 | 198 | hypothetical protein | hypothetical protein P108_0028 [Staphylococcus phage P108] | 3,00E-143 | |
| 102 | 52,243..54,162 | - | 1920 | 73.29 | 4.89 | 639 | recombination exonuclease B | hypothetical protein P108_0027 [Staphylococcus phage P108] | 0,00E+00 | AAA_23 (PF13476) AAA domain; Rad50_zn_hook (PF04423) Rad50 zinc hook motif |
| 103 | 54,162..54,539 | - | 378 | 15.17 | 5.32 | 125 | hypothetical protein | hypothetical protein P108_0026 [Staphylococcus phage P108] | 7,00E-86 | |
| 104 | 54,539..55,564 | - | 1026 | 39.38 | 4.78 | 341 | recombination exonuclease A | hypothetical protein P108_0025 [Staphylococcus phage P108] | 0,00E+00 | Metallophos (PF00149) Calcineurin-like phosphoesterase; Metallophos_2 (PF12850) Calcineurin-like phosphoesterase superfamily domain |
| 105 | 55,564..55,983 | - | 420 | 16.16 | 5.22 | 139 | hypothetical protein | hypothetical protein [Staphylococcus phage SA3] | 1,00E-97 | |

| | | | | | | | | | | |
|---|---|---|---|---|---|---|---|---|---|---|
| 106 | 56,062..57,504 | - | 1443 | 54.57 | 5.56 | 480 | DNA helicase | DNA helicase [Staphylococcus phage P108] | 0,00E+00 | DnaB_C (PF03796) DnaB-like helicase C terminal domain; AAA_25 (PF13481) AAA domain; ATPase (PF06745) KaiC |
| 107 | 57,497..59,11 | - | 1614 | 63.14 | 8.38 | 537 | Rep protein | putative Rep protein [Staphylococcus phage P108] | 0,00E+00 | |
| 108 | 59,122..60,87 | - | 1749 | 67.28 | 5.87 | 582 | DNA helicase A | DNA helicase [Staphylococcus phage SA3] | 0,00E+00 | Helicase_C (PF00271) Helicase conserved C-terminal domain; ResIII (PF04851) Type III restriction enzyme, res subunit |
| 109 | 60,96..62,336 | - | 1377 | 50.64 | 6.41 | 458 | tail fiber protein | putative tail fiber protein [Staphylococcus phage P108] | 0,00E+00 | |
| 110 | 62,343..62,717 | - | 375 | 14.64 | 4.46 | 124 | hypothetical protein | hypothetical protein P108_0018 [Staphylococcus phage P108] | 5,00E-86 | DUF2977 (PF11192) Protein of unknown function (DUF2977) |
| 111 | 62,731..64,653 | - | 1923 | 72.60 | 6.95 | 640 | hypothetical protein | hypothetical protein P108_0017 [Staphylococcus phage P108] | 0,00E+00 | CBM_4_9 (PF02018) Carbohydrate binding domain |
| 112 | 64,654..64,812 | - | 159 | 6.31 | 9.01 | 52 | hypothetical protein | hypothetical protein P108_0016 [Staphylococcus phage P108] | 1,00E-27 | |
| 113 | 64,861..68,319 | - | 3459 | 129.23 | 4.80 | 1152 | adsorption-associated tail protein | adsorption-associated tail protein [Staphylococcus phage P108] | 0,00E+00 | DUF4815 (PF16075) Domain of unknown function (DUF4815) |
| 114 | 68,34..68,861 | - | 522 | 19.25 | 5.04 | 173 | baseplate morphogenetic protein | hypothetical protein P108_0014 [Staphylococcus phage P108] | 3,00E-123 | |
| 115 | 68,972..72,037 | - | 3066 | 116.40 | 4.64 | 1021 | tail morphogenetic protein | hypothetical protein P108_0013 [Staphylococcus phage P108] | 0,00E+00 | |
| 116 | 72,058..73,104 | - | 1047 | 39.21 | 4.48 | 348 | baseplate morphogenetic protein | putative tail protein [Staphylococcus phage P108] | 0,00E+00 | Baseplate_J (PF04865) Baseplate J-like protein |
| 117 | 73,119..73,823 | - | 705 | 26.59 | 4.38 | 234 | baseplate wedge subunit | ORF052 [Staphylococcus virus G1] | 3,00E-172 | DUF2634 (PF10934) Protein of unknown function (DUF2634) |
| 118 | 73,823..74,347 | - | 525 | 20.02 | 4.28 | 174 | hypothetical protein | hypothetical protein [Staphylococcus phage SA3] | 2,00E-122 | |

| | | | | | | | | | | |
|---|---|---|---|---|---|---|---|---|---|---|
| 119 | 74,347..75,138 | - | 792 | 29.33 | 8.64 | 263 | hypothetical protein | hypothetical protein P108_0008 [Staphylococcus phage P108] | 0,00E+00 | |
| 120 | 75,245..77,791 | - | 2547 | 96.05 | 4.57 | 848 | glycerophosphoryl diester phosphodiesterase | putative glycerophosphoryl diester phosphodiesterase [Staphylococcus phage P108] | 0,00E+00 | GDPD (PF03009) Glycerophosphoryl diester phosphodiesterase family |
| 121 | 77,791..78,678 | - | 888 | 34.53 | 4.17 | 295 | peptidoglycan hydrolase | hypothetical protein P108_0006 [Staphylococcus phage P108] | 0,00E+00 | |
| 122 | 78,692..81,118 | - | 2427 | 91.31 | 6.40 | 808 | tail murein hydrolase | N-acetylmuramoyl-L-alanine amidase [Staphylococcus phage SA3] | 0,00E+00 | CHAP (PF05257) CHAP domain |
| 123 | 81,196..85,254 | - | 4059 | 143.86 | 9.72 | 1352 | tail tape measure | hypothetical protein [Staphylococcus phage SA3] | 0,00E+00 | |
| 124 | 85,307..85,843 | - | 537 | 20.98 | 4.02 | 178 | tail morphogenetic protein | tail morphogenetic protein [Staphylococcus phage P108] | 4,00E-127 | |
| 125 | 85,887..86,345 | - | 459 | 18.11 | 4.42 | 152 | hypothetical protein | hypothetical protein GH15_105 [Staphylococcus phage GH15] | 3,00E-106 | |
| 126 | 86,477..86,788 | - | 312 | 12.24 | 5.94 | 103 | hypothetical protein | hypothetical protein GH15_104 [Staphylococcus phage GH15] | 7,00E-67 | |
| 127 | 86,859..87,053 | - | 195 | 7.34 | 10.21 | 64 | hypothetical membrane protein | hypothetical protein P108_0223 [Staphylococcus phage P108] | 7,00E-34 | |
| 128 | 87,066..87,518 | - | 453 | 17.81 | 10.29 | 150 | hypothetical protein | hypothetical protein GH15_103 [Staphylococcus phage GH15] | 2,00E-105 | |
| 129 | 87,561..87,701 | - | 141 | 5.44 | 11.42 | 46 | hypothetical protein | hypothetical protein [Staphylococcus phage [illegible] | 2,00E-23 | |
| 130 | 87,691..87,849 | - | 159 | 6.47 | 10.63 | 52 | hypothetical protein | hypothetical protein GH15_101 [Staphylococcus phage GH15] | 4,00E-29 | |
| 131 | 87,939..88,367 | - | 429 | 15.93 | 5.25 | 142 | tail tube protein | ORF105 [Staphylococcus virus G1] | 9,00E-101 | |
| 132 | 88,44..90,203 | - | 1764 | 64.47 | 4.59 | 587 | major tail sheath protein | putative major tail sheath protein [Staphylococcus phage P108] | 0,00E+00 | |
| 133 | 90,23..90,445 | - | 216 | 8.28 | 8.91 | 71 | hypothetical protein | ORF202 [Staphylococcus virus G1] | 4,00E-46 | |
| 134 | 90,447..91,283 | - | 837 | 31.76 | 4.41 | 278 | hypothetical protein | hypothetical protein GH15_097 [Staphylococcus phage GH15] | 0,00E+00 | |
| 135 | 91,302..91,922 | - | 621 | 23.75 | 11.09 | 206 | hypothetical protein | hypothetical protein GH15_096 [Staphylococcus phage GH15] | 2,00E-149 | |
| 136 | 91,922..92,8 | - | 879 | 33.71 | 5.52 | 292 | capsid protein | capsid protein [Staphylococcus phage JD007] | 0,00E+00 | |
| 137 | 92,814..93,722 | - | 909 | 34.17 | 4.89 | 302 | hypothetical protein | ORF030 [Staphylococcus virus G1] | 0,00E+00 | |
| 138 | 93,735..94,031 | - | 297 | 11.23 | 10.12 | 98 | hypothetical protein | hypothetical protein GH15_093 [Staphylococcus phage GH15] | 7,00E-61 | |

| | | | | | | | | | | |
|---|---|---|---|---|---|---|---|---|---|---|
| 139 | 94,123..95,514 | - | 1392 | 51.22 | 4.80 | 463 | major capsid protein | putative capsid protein [Staphylococcus phage GH15] | 0,00E+00 | |
| 140 | 95,63..96,589 | - | 960 | 36.17 | 4.07 | 319 | hypothetical protein | hypothetical protein qdsa002_106 [Staphylococcus phage qdsa002] | 0,00E+00 | |
| 141 | 96,608..97,381 | - | 774 | 28.65 | 4.64 | 257 | prohead protease | ORF048 [Staphylococcus virus G1] | 0,00E+00 | Peptidase_S78 (PF04586) Caudovirus prohead serine protease Peptidase_S78 (PF04586) |
| 142 | 97,574..99,265 | - | 1692 | 64.10 | 6.56 | 563 | portal protein | putative portal protein [Staphylococcus phage GH15] | 0,00E+00 | Phage_portal (PF04860) Phage portal protein |
| 143 | 99,269..99,64 | - | 372 | 14.51 | 5.78 | 123 | hypothetical protein | ORF115 [Staphylococcus virus G1] | 2,00E-83 | |
| 144 | 99,659..99,847 | - | 189 | 7.17 | 9.66 | 62 | hypothetical membrane protein | putative membrane protein [Staphylococcus phage GH15] | 2,00E-36 | |
| 145 | 100,085..101,236 | - | 1152 | 42.05 | 3.83 | 383 | hypothetical protein | putative membrane protein [Staphylococcus phage phiSA12] | 0,00E+00 | |
| 146 | 101,329..101,808 | - | 480 | 18.53 | 4.53 | 159 | hypothetical protein | ORF091 [Staphylococcus virus G1] | 2,00E-110 | |
| 147 | 101,805..101,978 | - | 174 | 6.69 | 9.93 | 57 | hypothetical membrane protein | hypothetical protein [Staphylococcus phage [illegible] | 2,00E-30 | |
| 148 | 101,965..102,765 | - | 801 | 29.87 | 5.03 | 266 | hypothetical protein | hypothetical protein 812_087 [Staphylococcus phage 812] | 0,00E+00 | |
| 149 | 102,779..104,596 | - | 1818 | 70.25 | 6.87 | 605 | terminase large subunit | ORF010 [Staphylococcus virus G1] | 0,00E+00 | Terminase_GpA (PF05876) Phage terminase large subunit (GpA) |
| 150 | 104,611..105,021 | - | 411 | 15.65 | 4.11 | 136 | hypothetical protein | ORF133 [Staphylococcus virus G1] | 2,00E-92 | |
| 151 | 105,018..105,296 | - | 279 | 10.58 | 10.23 | 92 | hypothetical protein | ORF161 [Staphylococcus virus G1] | 2,00E-61 | |
| 152 | 105,274..105,54 | - | 267 | 10.34 | 8.85 | 88 | hypothetical protein | ORF168 [Staphylococcus virus G1] | 7,00E-55 | |
| 0152A | 105,801..106,067 | + | 267 | 10.12 | 9.71 | 88 | hypothetical membrane protein | ORF169 [Staphylococcus virus G1] | 7,00E-51 | |
| 153 | 106,1..106,426 | + | 327 | 13.08 | 5.67 | 108 | hypothetical membrane protein | putative membrane protein [Staphylococcus phage GH15] | 8,00E-72 | |
| 154 | 106,439..106,771 | + | 333 | 12.50 | 4.99 | 110 | hypothetical protein | hypothetical protein [Staphylococcus phage [illegible] | 4,00E-70 | |
| 155 | 106,784..106,993 | + | 210 | 8.02 | 5.78 | 69 | hypothetical protein | ORF207 [Staphylococcus virus G1] | 4,00E-43 | |
| 156 | 107,484..107,702 | + | 219 | 8.68 | 9.38 | 72 | Iro | ORF200 [Staphylococcus virus G1] | 6,00E-46 | |
| 157 | 109,248..109,433 | + | 186 | 7.07 | 4.72 | 61 | hypothetical protein | ORF233 [Staphylococcus virus G1] | 2,00E-34 | |
| 158 | 109,518..110,021 | + | 504 | 18.10 | 3.85 | 167 | holin | phage philc3 family protein [Staphylococcus phage JD007] | 3,00E-117 | Phage_holin_1 (PF04531) Bacteriophage holin |

| | | | | | | | | | | |
|---|---|---|---|---|---|---|---|---|---|---|
| 159 | 110,021..111,511 | + | 1491 | 54.74 | 10.08 | 496 | endolysin | endolysin [Staphylococcus phage phiIPLA-RODI] | 0,00E+00 | SH3_5 (PF08460) Bacterial SH3 domain; CHAP (PF05257) CHAP domain; Amidase_2 (PF01510) N-acetylmuramoyl-L-alanine amidase |
| 160 | 111,574..112,194 | + | 621 | 24.61 | 9.52 | 206 | hypothetical protein | hypothetical protein [Staphylococcus phage MCE-2014] | 4,00E-146 | |
| 161 | 112,308..112,616 | + | 309 | 12.21 | 9.73 | 102 | hypothetical membrane protein | hypothetical protein vBSauCG_215 [Staphylococcus phage vB_Sau_CG] | 8,00E-55 | |
| 162 | 112,616..113,407 | + | 792 | 29.37 | 9.40 | 263 | hypothetical membrane protein | ORF044 [Staphylococcus virus G1] | 0,00E+00 | Band_7 (PF01145) PFH domain / Band 7 family |
| 163 | 113,474..114,109 | + | 636 | 24.83 | 9.439 | 211 | hypothetical protein | ORF058 [Staphylococcus virus G1] | 1,00E-152 | |
| 164 | 114,296..114,988 | + | 693 | 24.88 | 4.77 | 230 | transglycosylase | putative transglycosylase [Staphylococcus phage phiSA12] | 2,00E-166 | |
| 165 | 115,097..115,324 | + | 228 | 9.23 | 10.61 | 75 | hypothetical protein | ORF190 [Staphylococcus virus G1] | 1,00E-45 | |
| 166 | 115,327..115,557 | + | 231 | 8.83 | 9.79 | 76 | transcriptional regulator | transcriptional regulator [Staphylococcus phage JD007] | 2,00E-47 | HTH_3 (PF01381) Helix-turn-helix |
| 167 | 115,547..116,188 | + | 642 | 24.59 | 3.81 | 213 | hypothetical protein | ORF057 [Staphylococcus virus G1] | 2,00E-144 | |
| 168 | 116,211..116,402 | + | 192 | 7.47 | 5.79 | 63 | hypothetical protein | ORF222 [Staphylococcus virus G1] | 6,00E-38 | |
| 169 | 116,392..116,817 | + | 426 | 15.81 | 7.40 | 141 | ribonuclease H | ORF096 [Staphylococcus virus G1] | 2,00E-95 | RNase_H (PF00075) Rnase H; RVT_3 (PF13456) - transcriptase-like |
| 170 | 116,833..117,447 | + | 615 | 23.02 | 3.82 | 204 | hypothetical protein | ORF063 [Staphylococcus virus G1] | 4,00E-145 | |
| 171 | 117,499..118,239 | + | 741 | 28.59 | 4.93 | 246 | PhoH-related protein | ORF049 [Staphylococcus virus G1] | 0,00E+00 | PhoH (PF02562) PhoH-like protein |
| 172 | 118,308..118,532 | + | 225 | 8.17 | 9.10 | 74 | hypothetical membrane protein | hypothetical protein [Staphylococcus phage [illegible] | 1,00E-40 | |
| 173 | 118,532..119,428 | + | 897 | 35.03 | 5.25 | 298 | DNA ligase | DNA ligase [Staphylococcus phage JD007] | 0,00E+00 | RNA_ligase (PF09414) RNA ligase |
| 174 | 119,421..120,014 | + | 594 | 22.26 | 4.56 | 197 | nucleoside 2-deoxyribosyltransferase | hypothetical protein [Staphylococcus phage MCE-2014] | 8,00E-144 | |
| 175 | 120,007..120,585 | + | 579 | 21.38 | 8.58 | 192 | hypothetical membrane protein | hypothetical protein [Staphylococcus phage MCE-2014] | 5,00E-131 | |
| 176 | 120,592..120,765 | + | 174 | 6.69 | 7.35 | 57 | hypothetical protein | hypothetical protein [Staphylococcus phage JD007] | 2,00E-32 | LysM (PF01476) LysM domain |

| | | | | | | | | | | |
|---|---|---|---|---|---|---|---|---|---|---|
| 177 | 120,782..121,045 | + | 264 | 10.23 | 4.89 | 87 | hypothetical protein | hypothetical protein P108_0172 [Staphylococcus phage P108] | 3,00E-55 | |
| 178 | 121,124..123,172 | + | 2049 | 79.71 | 6.65 | 682 | hypothetical protein | hypothetical protein phi_676Z_ORF036 [Staphylococcus phage 676Z] | 0,00E+00 | PcfJ (PF14284) PcfJ-like protein |
| 179 | 123,172..123,333 | + | 162 | 6.37 | 4.35 | 53 | hypothetical protein | ORF259 [Staphylococcus virus G1] | 7,00E-30 | |
| 180 | 123,377..123,565 | + | 189 | 7.30 | 4.03 | 62 | hypothetical protein | hypothetical protein phi_A3R_ORF034 [Staphylococcus phage A3R] | 8,00E-35 | |
| 181 | 123,565..123,867 | + | 303 | 11.31 | 4.53 | 100 | hypothetical protein | ORF149 [Staphylococcus virus G1] | 7,00E-65 | MazG (PF03819) MazG nucleotide pyrophosphohydrolase domain |
| 182 | 124,001..124,417 | + | 417 | 15.98 | 4.84 | 138 | hypothetical protein | ORF106 [Staphylococcus virus G1] | 6,00E-96 | DUF3310 (PF11753) Protein of unknwon function (DUF3310) |
| 183 | 124,41..124,736 | + | 327 | 12.98 | 4.41 | 108 | hypothetical protein | ORF134 [Staphylococcus virus G1] | 3,00E-73 | |
| 184 | 124,888..126,006 | + | 1119 | 42.22 | 4.47 | 372 | AAA family ATPase | ORF024 [Staphylococcus virus G1] | 0,00E+00 | AAA_5 (PF07728) AAA domain (dynein-related subfamily) |
| 185 | 126,018..126,863 | + | 846 | 31.77 | 9.64 | 281 | hypothetical protein | ORF038 [Staphylococcus virus G1] | 0,00E+00 | |
| 186 | 126,863..127,138 | + | 276 | 10.86 | 4.68 | 91 | hypothetical membrane protein | ORF163 [Staphylococcus virus G1] | 9,00E-54 | MFS_Mycoplasma (PF07672) Mycoplasma MFS transporter |
| 187 | 127,141..127,305 | + | 165 | 6.26 | 10.22 | 54 | hypothetical membrane protein | ORF256 [Staphylococcus virus G1] | 2,00E-29 | |
| 188 | 127,308..127,841 | + | 534 | 20.81 | 4.11 | 177 | hypothetical protein | ORF077 [Staphylococcus virus G1] | 1,00E-122 | |
| 189 | 127,841..128,383 | + | 543 | 20.34 | 4.57 | 180 | hypothetical protein | ORF072 [Staphylococcus virus G1] | 3,00E-122 | |
| 190 | 128,433..128,915 | + | 483 | 18.84 | 4.26 | 160 | hypothetical protein | ORF090 [Staphylococcus virus G1] | 8,00E-111 | |
| 191 | 128,956..129,129 | + | 174 | 6.81 | 4.92 | 57 | hypothetical protein | ORF245 [Staphylococcus virus G1] | 8,00E-35 | |
| 192 | 129,228..129,617 | + | 390 | 15.15 | 4.63 | 129 | hypothetical protein | ORF114 [Staphylococcus virus G1] | 4,00E-89 | |
| 193 | 129,619..129,858 | + | 240 | 9.35 | 4.39 | 79 | hypothetical protein | gpORF020 [Staphylococcus phage A5W] | 3,00E-51 | |
| 0193A | 129,87..129,974 | + | 105 | 4.11 | 4.45 | 34 | hypothetical protein | ORF437 [Staphylococcus virus G1] | 1,00E-14 | |
| 194 | 130,037..130,774 | + | 738 | 28.67 | 6.54 | 245 | hypothetical protein | ORF050 [Staphylococcus virus G1] | 3,00E-175 | |
| 195 | 130,764..130,958 | + | 195 | 7.64 | 4.32 | 64 | hypothetical protein | ORF218 [Staphylococcus virus G1] | 2,00E-38 | |
| 196 | 130,959..131,177 | + | 219 | 8.43 | 4.14 | 72 | hypothetical protein | ORF201 [Staphylococcus virus G1] | 9,00E-44 | |

| | | | | | | | | | | |
|---|---|---|---|---|---|---|---|---|---|---|
| 197 | 131,181..131,729 | + | 549 | 21.95 | 4.18 | 182 | hypothetical protein | ORF071 [Staphylococcus virus G1] | 9,00E-125 | |
| 0197A | 131,945..132,145 | + | 201 | 7.27 | 10.24 | 66 | hypothetical protein | hypothetical protein qdsa002_45 [Staphylococcus phage qdsa002] | 8,00E-41 | |
| 198 | 132,715..133,032 | + | 318 | 11.84 | 6.87 | 105 | hypothetical protein | ORF138 [Staphylococcus virus G1] | 6,00E-68 | |
| 199 | 133,048..133,602 | + | 555 | 21.22 | 4.29 | 184 | hypothetical protein | hypothetical protein [Staphylococcus phage phiIPLA-RODI] | 9,00E-131 | |
| 200 | 133,702..134,409 | + | 708 | 27.63 | 4.85 | 235 | serine/threonine protein phosphatase | phosphatase [Staphylococcus phage phiIPLA-RODI] | 5,00E-172 | Metallophos (PF00149) Calcineurin-like phosphoesterase; Metallophos_2 (PF12850) Calcineurin-like phosphoesterase superfamily domain |
| 201 | 134,406..134,804 | + | 399 | 16.11 | 9.35 | 132 | hypothetical protein | hypothetical protein [Staphylococcus phage S25-[illegible] | 7,00E-89 | |
| 202 | 134,817..135,305 | + | 489 | 19.53 | 10.07 | 162 | hypothetical protein | hypothetical protein [Staphylococcus phage phiIPLA-RODI] | 2,00E-117 | DUF1768 (PF08719) Domain of unknown function (DUF1768) |
| 203 | 135,317..135,859 | + | 543 | 21.56 | 9.85 | 180 | nucleotidyl transferase | nucleotidyl transferase [Staphylococcus phage phiIPLA-RODI] | 1,00E-125 | |
| 204 | 135,873..136,304 | + | 432 | 16.75 | 3.93 | 143 | hypothetical protein | hypothetical protein [Staphylococcus phage phiIPLA-RODI] | 1,00E-98 | |
| 205 | 136,297..136,782 | + | 486 | 18.36 | 9.85 | 161 | hypothetical membrane protein | membrane protein [Staphylococcus phage phiIPLA-RODI] | 3,00E-109 | |
| 0205A | 136,779..136,97 | + | 192 | 7.88 | 10.37 | 63 | hypothetical membrane protein | hypothetical protein [Staphylococcus phage [illegible] | 7,00E-37 | |
| 206 | 136,973..137,404 | + | 432 | 17.25 | 4.23 | 143 | UboA | UboA [Staphylococcus phage phiIPLA-RODI] | 6,00E-96 | |
| 207 | 137,404..137,811 | + | 408 | 16.47 | 5.03 | 135 | hypothetical protein | ORF109 [Staphylococcus virus G1] | 3,00E-94 | |
| 208 | 137,824..138,309 | + | 486 | 19.12 | 5.51 | 161 | hypothetical protein | hypothetical protein [Staphylococcus phage phiIPLA-RODI] | 2,00E-108 | |
| 209 | 138,311..138,547 | + | 237 | 9.56 | 4.52 | 78 | BofL | hypothetical protein CPT_phageK_gp022 [Staphylococcus virus K] | 5,00E-50 | |
| 210 | 138,651..138,941 | + | 291 | 11.60 | 4.23 | 96 | hypothetical protein | hypothetical protein [Staphylococcus phage phiIPLA-RODI] | 5,00E-64 | |
| 0210A | 139,094..139,195 | - | 102 | 3.82 | 8.91 | 33 | hypothetical protein | ORF450 [Staphylococcus virus G1] | 6,00E-14 | |
| 211 | 139,269..139,49 | - | 222 | 8.70 | 4.56 | 73 | TreU | hypothetical protein CPT_phageK_gp021 [Staphylococcus virus K] | 7,00E-43 | |

| | | | | | | | | | |
|---|---|---|---|---|---|---|---|---|---|
| 212 | 139,575..139,844 | - | 270 | 10.24 | 6.80 | 89 | TreT | ORF166 [Staphylococcus virus G1] | 4,00E-57 |
| 213 | 139,844..140,017 | - | 174 | 6.66 | 4.69 | 57 | TreS | ORF234 [Staphylococcus virus G1] | 5,00E-31 |
| 214 | 140,076..140,546 | - | 471 | 17.92 | 3.52 | 156 | hypothetical protein | ORF092 [Staphylococcus virus G1] | 6,00E-104 |
| 215 | 140,626..140,862 | - | 237 | 9.08 | 3.98 | 78 | hypothetical protein | hypothetical protein CPT_phageK_gp017 [Staphylococcus virus K] | 5,00E-47 |
| 216 | 140,953..141,117 | - | 165 | 6.38 | 4.39 | 54 | hypothetical protein | hypothetical protein [Staphylococcus phage [illegible] | 2,00E-28 |
| 217 | 141,188..141,325 | - | 138 | 5.45 | 4.13 | 45 | hypothetical protein | hypothetical protein [Staphylococcus phage [illegible] | 8,00E-25 |
| 218 | 141,406..141,627 | - | 222 | 8.47 | 3.91 | 73 | hypothetical protein | hypothetical protein GH15_008 [Staphylococcus phage GH15] | 1,00E-45 |
| 219 | 142,122..142,523 | - | 402 | 15.48 | 4.18 | 133 | hypothetical protein | hypothetical protein [Staphylococcus phage MCE-2014] | 5,00E-92 |
| 220 | 142,623..142,946 | - | 324 | 12.44 | 4.90 | 107 | TreP | hypothetical protein CPT_phageK_gp016 [Staphylococcus virus K] | 1,00E-69 |
| 221 | 143,114..143,245 | - | 132 | 5.15 | 10.67 | 43 | TreO | ORF297 [Staphylococcus virus G1] | 2,00E-21 |

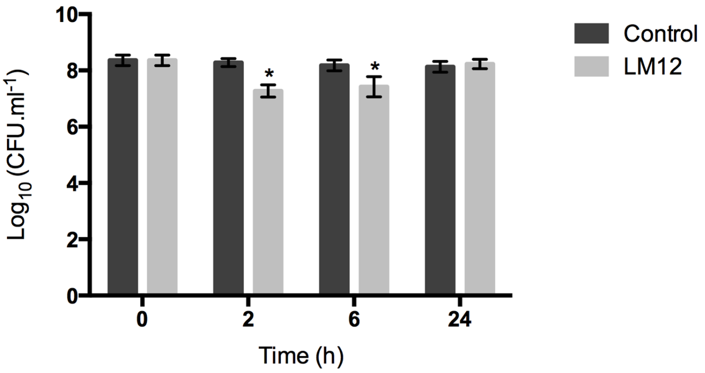


**Figure S1:** *Phage LM12 infection on 24 h S. aureus biofilms, using a MOI 1.* Data was assessed by colony forming units (CFU) counting and the values represent the mean plus and minus of three independent experiments performed in duplicate. Statistical differences ($p < 0.01$) between control biofilms and LM12-treated biofilms (*) were determined by two-way repeated-measures analysis of variance (ANOVA) with Sidak post test.

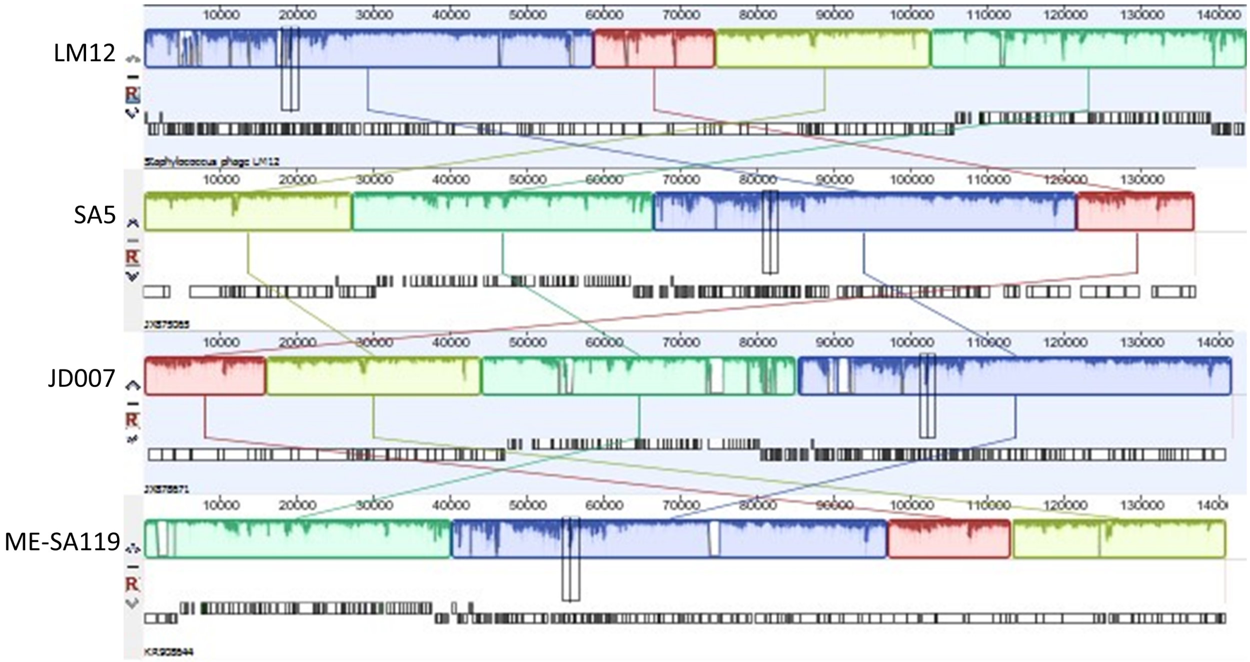


***Figure S2****: Multiple genome alignment of Staphylococcus phage LM12 with closest homologs.* The figure was performed using Geneious 9.1.4.
